# Calibrating Self-Reported Measures of Maternal Smoking in Pregnancy via Bioassays Using a Monte Carlo Approach

**DOI:** 10.3390/ijerph6061744

**Published:** 2009-06-03

**Authors:** Vanja M. Dukic, Marina Niessner, Kate E. Pickett, Neal L. Benowitz, Lauren S. Wakschlag

**Affiliations:** 1 Department of Health Studies, University of Chicago, USA; 2 Department of Economics, University of Chicago, USA; E-Mail: marinak@uchicago.edu; 3 Department of Health Sciences, University of York, UK; E-Mail: kp6@york.ac.uk; 4 Departments of Medicine, Psychiatry and Biopharmaceutical Sciences, University of California, San Francisco, USA; E-Mail: nbenowitz@medsfgh.ucsf.edu; 5 Institute for Juvenile Research, Department of Psychiatry, University of Illinois at Chicago, Chicago, IL USA; E-Mail: lwakschlag@psych.uic.edu

**Keywords:** smoking, self-report, bioassay, calibration

## Abstract

Maternal smoking during pregnancy is a major public health problem that has been associated with numerous short- and long-term adverse health outcomes in offspring. However, characterizing smoking exposure during pregnancy precisely has been rather difficult: self-reported measures of smoking often suffer from recall bias, deliberate misreporting, and selective non-disclosure, while single bioassay measures of nicotine metabolites only reflect recent smoking history and cannot capture the fluctuating and complex patterns of varying exposure of the fetus. Recently, Dukic *et al.* [1] have proposed a statistical method for combining information from both sources in order to increase the precision of the exposure measurement and power to detect more subtle effects of smoking. In this paper, we extend the Dukic *et al.* [1] method to incorporate individual variation of the metabolic parameters (such as clearance rates) into the calibration model of smoking exposure during pregnancy. We apply the new method to the Family Health and Development Project (FHDP), a small convenience sample of 96 predominantly workingclass white pregnant women oversampled for smoking. We find that, on average, misreporters smoke 7.5 cigarettes more than what they report to smoke, with about one third underreporting by 1.5, one third under-reporting by about 6.5, and one third underreporting by 8.5 cigarettes. Partly due to the limited demographic heterogeneity in the FHDP sample, the results are similar to those obtained by the deterministic calibration model, whose adjustments were slightly lower (by 0.5 cigarettes on average). The new results are also, as expected, less sensitive to assumed values of cotinine half-life.

## 1. Introduction

Maternal smoking during pregnancy is a major public health problem that has been associated with numerous short- and long-term adverse health outcomes in offspring [2–5]. Infants who were prenatally exposed to maternal cigarette smoke are at increased risk of low birth weight, preterm delivery, fetal growth restriction and perinatal mortality [2,6–8]. Although the adverse perinatal (e.g., premature birth, low birth weight) effects of maternal smoking and in-utero fetal exposure have been well-known for some time, a growing body of literature points to evidence for long-term and intergenerational health consequences for children. Furthermore, there is increasing evidence of longterm behavioral sequelae of exposure, with a robust association to conduct problems in offspring [9–11], including evidence of gene-exposure interaction [12,13]. Although maternal smoking has systematically declined over the 1990s, recent national vital statistics reports suggest that 12.3% of women still smoke during pregnancy [14]. Smoking prevalence varies widely by region, ethnicity, age and socioeconomic status, ranging from 3–42% [14,15]. In addition, pregnant women are increasingly more aware that smoking may be harmful to their offspring, and are also aware of social pressures to quit [16], so substantial fluctuations in smoking behavior can occur [17,18].

Assessing prenatal exposure to cigarette smoke, like assessing other addictive behaviors, is challenging. The existing methods imperfectly capture “true” exposure, which is the complex result of maternal behavior, smoking topography (dose) and metabolism. However, the accuracy of measurement is crucial for studies of long-term effects of exposure on behavior where effects are subtle, complex and long-term.

Smoking behavior during pregnancy has traditionally been assessed either through self-report or biologic measures of nicotine metabolites (such as cotinine). Self-report is efficient and low cost and it uniquely captures information about historical patterns of smoking. However, self-report is prone to biases such as under-reporting due to social pressures and recall bias [19]. Furthermore, variations in smoking topography will translate into different amounts of exposure since the same self-reported number of cigarettes smoked does not generally correspond to the same amount of inhaled smoke. On the other hand, biologic assays provide unique information about individual intake of smoking constituents and thus are generally considered more accurate measures of smoking. However, assays reflect exposure only over a relatively recent short period (three days or less in pregnancy [20]. Similarly, variations in nicotine metabolism across individuals and during pregnancy will result in elimination of nicotine and its metabolites at different rates, and consequently in different net exposure to fetus. Thus, since both self-report and bioassays contribute unique information and error to the assessment of exposure, combining the two is likely to enhance precision, and allow us to calibrate maternal self-report based on smoking topography and metabolism of cigarette smoke.

Recently Dukic *et al.* [1] proposed a “best estimate” method to mathematically combine self-reported and biologic exposure measures. This method describes the average relationship between urine cotinine (taking into account its exponential decay [20,21] and the number of reported cigarettes in a sample of pregnant women. Based on this average relationship, which is a combination of population characteristics and the characteristics of the sample, this model uses the cotinine measurements to ``probabilistically correct” the self-report. It also provides a classification of women into “reporting categories”, such as accurate reporters, underreporters, and extreme underreporters. Using this method in the Family Health and Development Project (FHDP), we found that on average, misreporters smoked about 8 cigarettes more than what they reported to smoke, with about one third underreporting by 1.5, one third under-reporting by about 6.5, and one third underreporting by 9 cigarettes [1]. While this method did allow us to calibrate the self-report, it did that using the same algorithm and metabolic parameters for all women. Given that women may differ in how quickly they metabolize nicotine, especially during pregnancy, deterministic method may be a bit too inflexible in its calibration. Uncertainty due to variation among women in the sample can be reflected in the final classifications and adjustments. Given that we are working with a non-linear relationship, this uncertainty could propagate into final classifications in non-obvious ways, appearing either as underor over-correction.

In this paper we thus propose an extension of the Dukic *et al.* [1] method for combining biological and self-reported measures that can also take into account the metabolic heterogeneity among women and over the course of the pregnancy. This extension allows us to capture mother-specific relationships between her own cotinine metabolism and self-report, which could change over trimesters, reflecting the accelerated metabolism during pregnancy. In other words, there are two sources of extra variability that the deterministic method did not account for: one is a woman’s own rate of nicotine metabolism, and the other is that rate of metabolism may vary in general for all women across the trimesters due to pregnancy. We use a Monte Carlo (MC) scheme to capture this additional randomness in a non-linear way, and obtain a better estimate of fetal exposure (and amount of misreporting) when metabolic differences exist.

## 2. Methods

*Data*. The data for this study are derived from The Family Health and Development Project (FHDP) (for details see [11,17,22]), a small convenience sample of 96 pregnant women oversampled for smoking and followed from early pregnancy through their infants’ 24-month birthdays. The sample consisted predominantly of non-Hispanic white, working-class pregnant women with high school education and low-to-moderate family income. Among them, 51 were non-smokers and 45 were smokers. All women were over 18 years of age, with the mean age among non-smokers of 28.9 years (standard deviation 5.6) and the mean age of smokers of 26.9 years (standard deviation 5.6). The study was approved by the IRB at the University of Chicago.

In this paper we analyze data only from those 45 women in FHDP sample who smoked during pregnancy. Most women had one visit per trimester. At each visit, urine samples were collected. Gas chromatography-mass spectrometry (GCMS) analyses were conducted to assess cotinine concentrations [23]. Maternal self-reported smoking was assessed at each visit in terms of weekday and weekend smoking in each month. These detailed self-report data were summarized by the mean daily number of cigarettes smoked in each trimester. In addition, self-reported number of cigarettes over each of the three days prior to the interview visit was recorded, as were the time of the interview and urine collection, and the mothers’ typical daily smoking pattern [19].

*Deterministic Method*. Under the assumptions of constant nicotine metabolism throughout pregnancy and no between-women metabolic differences (including similar amount, concentration and frequency of urination), Dukic *et al.* [1] were able to combine the self-report and cotinine smoking measures according to the deterministic laws of cotinine metabolism, and obtain a better overall measure of smoking exposure (the “corrected self-report”). However this simplifying homogeneity assumption may be unrealistic, as the variation among women and over time during pregnancy may be quite large.

More specifically, the method of Dukic *et al.* [1] is based on the mathematical model of decay of a nicotine metabolite (bioassay cotinine) in the body to adjust the self-report of smoking. The key idea in this model was the scale alignment. This involves first re-scaling the self-reported number of cigarettes so that they correspond to the same underlying scale as cotinine. This is because the cotinine in the body (and serum, saliva, or urine) reflects all cigarettes smoked over a certain period of time; however, not all cigarettes in that time period are equally represented. Since the amount of cotinine from each cigarette decays exponentially over time, the cigarettes smoked further in the past are not represented as heavily in the cotinine measure as those smoked more recently.

Thus, simply comparing the self-reported number of cigarettes (multiplied by a cotinine/cigarette conversion factor) to the direct cotinine reading would be erroneous, as they are not on the same scale. However, once the scales for the two measures are aligned, we can compare and combine them. The calibration of the self-report is then done based on the percentiles of the expected distribution of discrepancies between weighted self-report and cotinine.

*Monte Carlo Method*. Using a Monte Carlo method, we will extend the above approach to draw on: (a) individual mothers' patterns in repeated urine cotinine measurements over the course of pregnancy, (b) sample level data on the correspondence of self-reported smoking and cotinine levels, and, (c) information from independent experimental studies about the variability in the biological parameters driving the metabolism of nicotine during the course of the pregnancy. These three sources of information allow us to model woman-specific and trimester-specific factors (small but consistent deviations from the “average” metabolism over time). As a result, we will have a Monte Carlo sample of reporting classifications for each woman, reflecting the variation in classifications due to variations among women and over time.

Mathematically, the basic model is as follows. At any given point in time, a fraction of nicotine from each cigarette is converted into cotinine in the liver and released into the bloodstream. From there, a portion is excreted through the kidneys into urine. Once a woman voids, all the cotinine that has accumulated in her urine is expelled, and it starts to accumulate anew. The cotinine concentration in the blood decays exponentially with a median half-life of approximately nine hours [20], and after being filtered through the kidneys is deposited in the urine. Thus the amount of urine cotinine consists of what has been accumulated between the last time the woman voided and the time of the urine sample. Based on this, we can calculate the fraction (or mathematical “weight”) of cotinine from each particular cigarette present in the collected urine sample (assuming each cigarette is equal). This amount is the difference between the amount of cotinine that would have accumulated in the urine sample had the woman not voided at all previously, and the amount of cotinine from that cigarette which was accumulated up to, and then expelled at the time of her last voiding. This quantity depends on the half-life of cotinine decay in blood and the blood-urine conversion factor, as well as how recently the cigarette was smoked relative to the time of collection of the urine sample. All cigarette weights are added into the total weighted sum, resulting in the “net present value” of all cigarettes smoked over the three days prior to urine collection. As the last step, the measured cotinine concentration is converted into the number of these weighted cigarette equivalents, according to the steady state cotinine-per-cigarette ratio.

Dukic *et al.* [1] examined three different weighting scenarios based on how detailed the available information about timing of each cigarette was: (1) morning urine collection, with only average cigarettes per day reported; (2) urine collected during the interview with limited information about daily pattern of smoking; (3) urine collected during the interview using all information about pattern of daily smoking. They found little difference in the FHDP sample between the “uniform” cigarette smoking pattern (scenarios 1 and 2) and the actual reported daily pattern (scenario 3) based on the detailed FHDP survey. Detailed description of the algorithm used to derive the weights is available in the *Appendix* in [1]. Note that a substantial problem with this and many other datasets is the lack the actual times of voiding; it has been suggested by Dukic *et al.* [1] that in order to obtain more accurate alignment between urine-based biological and self-report measures in the future, the interviewers should record voiding times as well as smoking patterns of women in future studies. Serum and saliva cotinine measures should not suffer from this problem.

All aspects of cotinine metabolism in the model considered in [1] were assumed to be constant across women. While this was an improvement over single-measure approaches, it does not capture meaningful individual variation. One way to account for inter-woman variability (heterogeneity) is to allow each woman-specific parameter to come from a population distribution. This is what is commonly referred to as the random effect or multi-level modeling in statistical literature [24]. If there is heterogeneity present, classifications resulting from the random-effect model will properly reflect the overall uncertainty associated with the nicotine and cotinine metabolic processes, and will protect from over-correcting. However, random effect models can be sensitive to the specified distributional assumptions. Thus, we rely on the extensive expertise of the co-author on the metabolism of pregnancy smoking to elicit the distributional ranges for the woman-specific effects.

*Smoking metabolism parameters*. According to previous research done by Shiffman and colleagues [25], the average serum cotinine that is generated per cigarette smoked in general white and Hispanic population of light smokers is 12.5 ng/mL, also confirmed by studies of heavier smokers [26–29]. In addition, there is evidence that during pregnancy urinary cotinine levels are on average eight times higher than serum cotinine [29]. This implies about 100 ng/mL of urinary cotinine per cigarette. If we assume that every woman deviates from an “average woman” by a random amount due to unobserved differences in metabolism, smoking topography, and urination schedule, the resulting discrepancies between the self-report and biological measures will also be randomly distributed according to some resulting distribution. Our objective thus is to obtain this resulting distribution via a Monte Carlo simulation, and to base our classification probabilistically on where on this distribution each woman’s discrepancy lies with respect to the combined sample and expert information.

In addition to these individual woman-specific differences, it is reasonable to expect that metabolism changes over time during pregnancy. One theory postulates that the metabolism increases with time during pregnancy; Dukic *et al.* [1] have also observed an increasing trend in the average magnitude of discrepancies over time. We however do not measure these metabolic changes in our study, and thus the only way we could account for this variability is through a model which allows each trimester to have a trimester-specific effect. Thus, the woman-specific effects are designed to capture the effect of all unobserved differences among women’s metabolisms, body size, propensity to mis-report, but they stay the same for each woman over all three trimesters. The trimester effect on the other hand would be designed to reflect systematic differences due to average (across women) metabolic differences among trimesters. In order to account for both of these unobserved processes, we thus extend the Dukic *et al.* [1] model to include woman-specific effects (characterized by distributions of woman-specific cotinine half-lives and the extracted amounts of cotinine per cigarette), as well as trimester-specific effects.

The distribution of woman-specific cotinine half-lives is based on the data provided by the previous experiments and literature [20,26–29]: we treat the half-life time for each woman *i h_w_i_* _,_where (*i* = 1, ... ,45), from a normal distribution with mean 8.78 hours and standard deviation 1.67 hours. These data were taken from a study of nicotine and cotinine metabolism in pregnant women [20]. The trimester effect is assumed to modify each woman’s half-life parameter by a different amount in each trimester, but those amounts are assumed to be the same for all women. Due to lack of strong theory about shape of metabolic trends over pregnancy, we assume that these deviations are also normally distributed with a mean of zero, and the standard deviation equal to the standard deviation of the women effects. We denote the trimester effect by *h_t_j_* where *j* = 1, 2, 3, and then the cotinine half-life for a woman *i* in trimester *j* is obtained by adding the two effects*: h_w_ij_= h_w_i_ + h_t_j_*. We repeat this procedure 200 times, yielding 200 woman-trimester specific cotinine half-lives.

Next, in order to generate a woman-specific effect for the average amount of cotinine generated by a single cigarette, we generate a random variable *c_w_i_* by forming a product of two other normal variables: the blood-urine conversion factor generated from a Normal distribution with a mean of 8 and standard deviation of 0.5 (N(8,0.5)), and the serum-cotinine-per-cigarette from a Normal distribution with mean 12.5 and standard deviation of 2 (N(12.5,2)). These Normal distributions were chosen again based on expert advice, and they reflect the experimental ranges of the ratio of cotinine in urine to cotinine in blood, of and the cotinine per cigarette in blood, respectively. We assume again that trimester effects are normally distributed with mean zero and standard deviation equal to the standard deviation of woman-specific effects. The equality of standard deviation is somewhat arbitrary, but reflecting the belief that deviations across time are on the same scale as deviations from woman to woman. Again, we generate the cotinine per cigarette amount for a woman *i* in trimester *j* by adding her individual and trimester effect, like we did for half-lives.

After we generate the sample of 200 woman-trimester specific half-lives and the amounts of cotinine per cigarette, for each woman and for all three trimesters, we apply the Dukic *et al.* [1] classification thresholds to classify each woman based on each of the 200 differences between the weighted sum and cotinine-derived cigarettes. This way, for each woman we get 200 classifications into over-reporters, accurate reporters, under-reporters, and extreme under-reporters, in every trimester. We adopt a convention that if in any given trimester a woman has at least 50% classifications that are the same (over-reporter, accurate reporter, under-reporter or extreme underreporter), that category is chosen as that woman’s final classification for that trimester. If the classification is unclear, we would check whether the number of classifications that fall into the under and extreme under-reporter category jointly make up more than 50% of that woman’s classifications; if yes, we classify that woman as under-reporter, and if not, we classify her as an accurate reporter. (Note that unlike nondisclosure, over-reporting is rarely encountered in practice. But we still allow for this possibility, suspecting this classification is most likely due to deficient conversion of nicotine to cotinine, unusually rapid metabolism cotinine or very light smoking topography.)

## 3. Results: Classification and Calibration of Self-Report

The results of MC classifications of each woman in our sample, by trimester, and under each cigarette imputing scenario considered in [1], are summarized in Table 1. The results are very similar to the findings based on the original deterministic approach. In particular, the actual classifications are quite robust – only the average adjustments appear slightly larger in the Monte Carlo approach due to the non-linear nature of adjustments. Such small differences between the MC and deterministic methods are possibly due to the small sample size, as well as low demographic heterogeneity in the FHDP sample (mostly composed of white working-class younger women). Furthermore, as expected based on the findings of [1], all three scenarios are similar, in that the under-reporters seem to underreport between 6.5 and 8 cigarettes on average, while extreme under-reporters misreport by between 8 and 11 cigarettes. Little difference between the results obtained under scenarios 2 and 3 again implies that the self-reported pattern of smoking may not have much value in these analyses, likely because of so few smokers in the sample and the large uncertainty due to voiding and hydration schedules. We thus report only the results based on the Scenario 3 which takes advantage of the full daily smoking pattern data.

We also examine the consistency of classification status, defining consistency as “having the same status for all pregnancy visits”. Out of 45 smokers in the FHDP dataset, we find that slightly less than half of the women have a consistent classification status and 55% of women fluctuate in their classification across visits. Of the 45% who are consistent, 20% women are consistent extreme underreporters, 11% are consistent under-reporters, 14% consistently report accurately, 0% of women consistently over-report. Table 2 summarizes the classification of each woman in each trimester, her average trimester self-report, cotinine, and finally the corrected trimester average, using the weighting schemes from the third scenario.

If it is desired to use a more robust adjustment method, as in Dukic *et al.* [1] we show a way to correct for under-reporting (or over-reporting) based on the average of discrepancies of all women in their corresponding class for that trimester. For example, for a woman “X” who is an under-reporter in trimester *j*, if the average discrepancy between cotinine-based and self-reported cigarettes for all under-reporters in that trimester is 5, the correction would add to the self-report of woman X a term based on those 5 cigarettes. The averages of all correction amounts within each of the categories are summarized in Table 3.

### 3.1. Sensitivity Analysis

We now examine how much our results would change if we consider alternative distributions in our Monte Carlo scheme. Although the distributions we used were based on extensive expertise of one of the co-authors in modeling the metabolism of pregnancy smoking, and on the review of literature [25–29], the fact that little is known about cotinine metabolism during pregnancy prompts us to perform a sensitivity analysis to a range of average values for cotinine half-life and the amount of urinary cotinine produced by a cigarette. In particular, the observed ratios of cotinine level and weighted self-report (the actual numbers we would get if we assume self-report is correct and cigarettes are uniformly distributed) in our data set are quite variable (they are summarized for each category in each trimester in Table 4): the lowest ratio for accurate reporters is 201 ng/mL, which is almost twice the amount of cotinine per cigarette per mL of urine usually assumed. This discrepancy may imply that the women in our sample are under-reporting routinely, or that the parameter values we use are perhaps not the most appropriate ones for pregnancy metabolism. To address this, we varied the mean for cotinine concentration per cigarette (originally assumed 100 ng/mL), and mean half-life of cotinine (originally assumed 9 hours) to see how much better or worse the agreement between self-report and cotinine would get.

Changing the average half-life of urinary cotinine had almost no impact on the classification (see Table 5). However, adjusting the mean of urinary cotinine a cigarette produces did. As shown in Table 6, using 100 ng/mL (corresponding roughly to using the means 12.5 and 8 for blood cotinine per cigarette concentration and blood-to-urine conversion factor distributions, respectively) 29 out of 128 (22.7 %) women’s observations are classified as either over-reporters or accurate reporters. Using 150 ng/mL (corresponding approximately to the means 12.5 for blood cotinine and 12 for urine conversion factor), all of women’s observations that were classified as under-reporters before are now classified as either accurate or even in some cases over-reporters. Assuming that a cigarette produces 300 ng/mL (means 12.5 for the blood cotinine and 24 for urine conversion factor) urinary cotinine, 75% of visits get classified as either over or accurate reporters. Thus using 150 ng/mL seems to give us overall the most balanced results. Clearly, expert opinion and experimental information as summarized by the Monte Carlo distributions are crucial for accuracy in calibration studies such as ours. We carry out this sensitivity analysis simply to underline the delicate balance between self-report and cotinine and the natural underlying variability. Identical findings were obtained in the sensitivity analysis in the deterministic approach of Dukic *et al.* [1].

## 4. Discussion

We present a new approach for calibrating self-reported smoking using biologic assays of cotinine, based on deviations from what is considered to be an average cotinine amount per cigarette in urine over the course of pregnancy [20], while integrating multiple sources of information and heterogeneity among women with respect to exposure metabolism. We have extended the methods from Dukic *et al.* [1] to account for heterogeneity of women and provide classification and adjustment under uncertainty. The uncertainty in non-linear models, such as this one, not only result in larger variation in the results, but may also change the average values used to adjust the self-reports.

The MC method is similar in spirit to the method of Dukic *et al.* [1]. The main difference between the two is that the current method results in the Monte Carlo sample of classifications for each woman, rather than a single deterministic classification. This sample of classifications reflects the metabolic variability in each trimester for every woman. The Monte Carlo approach thus allows us (1) to determine how much the adjustment of self-report will vary once the heterogeneity among women (both in terms of metabolic quantities and trimester) is accounted for, and (2) to perform the most likely classification under conditions of metabolic uncertainty. The new MC method is expected to be more stable than the original method – a larger percentage of women would be classified as accurate reporters since the added variability protects from possible overcorrecting of self-reported measures over time.

Besides accounting for heterogeneity in metabolic parameters over time during pregnancy, there are still possible improvements to our approach. For example, systematic differences in times of urination and fluid intake (and thus urine volume) among women remain an important source of variability which could confound the effect of metabolic differences among women. Thus, future studies should pay particular attention to gathering information on these habits along with the biological measurements such as cotinine when trying to assess smoking exposure. If this could be done, our methods could be extended in a straight-forward manner to accommodate this extra information and yield more precise woman-specific adjustments.

In spite of the limited size, the FHDP study with its rich smoking exposure component has provided us with a unique opportunity to examine some of these questions about the relationship between self-report and biological measures. However, it is clear that small sample size is a limitation; in particular, studies with only one visit during pregnancy which has both self-report and urinary measurement would not provide good insight into the true smoking behavior over time during pregnancy. Although data on pregnant smokers are in general hard to collect, a larger and better designed dataset with additional repeated measurements of self-report and serum or urinary cotinine, along with detailed timing of voiding during the past three days, all on a larger sample, would almost certainly be very informative. For example, additional repeated measurements would give us a better sense of the pattern of the metabolism heterogeneity over time. In addition, in a sample with larger demographic and metabolic heterogeneity (due for example to a wider distribution in age, BMI, or greater genetic diversity), it is likely that much more variability over time and between women would be captured by the MC method. We hope that this novel approach to classification and calibration of self-reports based on biological measures can shed new light on measurement of smoking exposure in general, and not just during pregnancy, as they are applied to larger datasets.

There are several other limitations to our approach. One is the inability to account for individual variation in smoking topography, that is, how much nicotine is absorbed systemically from each cigarette. Environmental tobacco smoke exposure might also be considered a potential confounder, although cotinine levels from this source are usually insignificant compared to active smoking. Thus there are important potential confounders with metabolic differences, which will remain until better data on them are collected in future studies. Indeed, it may seem that the wide range of discrepancies between common assumptions and our findings implies that either most of the mothers in our rather homogenous sample are inaccurate reporters, or that the assumptions such as blood-urine cotinine conversion factor or cotinine per cigarette levels are simply not appropriate. We unfortunately cannot separate the assumptions from misreporting given our data without relying on further assumptions. More elaborate studies about smoking topography, as well as cotinine metabolism and excretion during pregnancy are needed to make progress with respect to this issue.

The methods proposed in this paper are another step towards more sophisticated methodology for integrating self-reported and biologic estimates of exposure, which are not only able to incorporate heterogeneity among women but also various sources of uncertainty in measurements and data collection. Estimating exposure more precisely enhances the capacity for rigorously testing a variety of hypotheses, as well as time and threshold effects, all of which are critical to establishing causality in exposure-behavior relationship. In future studies, we plan to test whether the combined method proposed here adds incremental value to prediction of behavioral outcomes in exposed offspring, over and above that based on self-report and/or cotinine alone.

## Figures and Tables

**Table 1.** Classification of women based on the individual MC corrections. For each category, the first column (“n”) is the number of women in that classification group. The second column (“mean”) is the mean of the differences between the weighted self-report and the number of cigarettes from cotinine. The third column (“sd”) is the standard deviation of these differences.

***Scen.***	***Trim.***	***Category***											
		***over reporting***			***accurate reporting***			***under reporting***			***extreme under reporting***		
		***freq***	***mean***	***sd***	***n***	***mean***	***sd***	***n***	***mean***	***sd***	***n***	***mean***	***sd***
1	1	-	-	-	13	−1.39	3.24	12	−7.92	5.31	13	−8.81	6.18
1	2	2	2.55	1.98	18	−1.06	2.22	13	−6.62	3.37	12	−8.42	4.34
1	3	-	-	-	15	−0.99	1.67	11	−5.85	3.16	15	−8.27	5.81
2	1	-	-	-	9	−2.28	1.76	14	−7.51	5.23	15	−10.15	6.34
2	2	-	-	-	14	−1.84	2.71	17	−6.80	3.70	14	−9.52	4.83
2	3	-	-	-	11	−2.13	1.79	13	−6.47	3.77	17	−8.69	5.96
3	1	-	-	-	10	−1.90	1.78	16	−6.48	4.71	12	−11.24	6.44
3	2	1	3.26	-	17	−1.95	2.15	15	−7.59	3.55	12	−8.88	4.76
3	3	-	-	-	13	−1.21	1.30	13	−7.13	3.80	15	−8.04	5.82

**Table 2.** Classification of women based on the individual MC corrections, under scenario 3. For each trimester, the first column (“Mean self-report”) shows the self-reported trimester daily average number of cigarettes. The second column (“Cot”) shows the cotinine level from the urine test in the given trimester. The third column (“Class”) shows the classification of the woman: O for over-reporter, A for accurate reporter, U for underreporter, and E for extreme under-reporter. The fourth column (“Corrected self-rep”) shows the corrected average trimester self-report. No adjustment could be calculated for women with missing cotinine measurements.

***ID***	***Trimester 1***				***Trimester 2***				***Trimester 3***			
	***Mean self-rep***	***Cot (ng/mL)***	***Class***	***Corrected self-rep***	***Mean self-rep***	***Cot (ng/mL)***	***Class***	***Corrected self-rep***	***Mean self-rep***	***Cot (ng/mL)***	***Class***	***Corrected self-rep***
1	10	509	A	13.29	8.67	543	A	11.38	10	1280	E	20.77
2	9.67	254	A	11.19	8	175	A	9.20	4	170	A	5.04
4	2	547	E	6.25	1	342	E	4.50	3	199	U	5.01
8	18.67	1130	U	26.97	20	1240	U	30.51	19	506	A	20.58
10	9.33	366	A	12.34	4	507	U	7.43	7	1230	E	19.01
11	13	1270	U	22.69	10	1840	E	26.76	12	2010	E	29.24
12	20	1670	U	31.61	20	1340	U	29.74	20			
13	0.09	61.5	E	0.54	0	0	A	0.00	0.5	57.9	E	1.19
19	13.33	1680	E	26.61	11.67	655	A	15.76	10	1020	U	16.99
20	13.67	1840	E	28.11	15	1570	U	27.73	14	776	U	19.90
22	8.67	769	U	13.78	13	784	U	18.21	7.5	550	A	7.79
26	10	1110	U	18.93	13.33	1260	U	23.65	15.5	997	U	21.36
31	0	33	U	0.38	0	10.7	A	0.11	0	61.6	U	0.69
35	6	1480	E	17.54	5	793	E	12.77	4.5	1130	E	13.28
38	0	596	E	6.05	0	632	E	6.31	0	319	E	3.23
41	20	266	A	18.75	23.33	704	A	26.63	20	490	A	22.44
47	10	1280	E	19.22	10	1100	U	17.15	10	991	U	16.42
48	9	1020	E	17.89	12.33	64.4	O	8.79	9	619	U	13.56
50	5	561	U	9.05	5	1480	E	18.42	8.5	714	U	13.72
53	13	587	A	15.94	16.67	692	A	21.08	19	1740	U	36.15
55	0.67	34.5	U	1.10	0.67	30.3	A	0.69	1.5	69	A	1.96
60	10				10	335	A	10.50	15	596	A	16.74
67	16.67	570	A	18.18	26.33	570	A	29.98	12.5	1020	U	21.15
68	8.67				2.67	565	E	6.76	0	1490	E	20.42
70	0				6.67	789	U	12.23	12.5	1020	U	21.04
74	8	322	A	10.46	8	618	U	12.97	8	420	A	7.63
75	10				10	870	U	16.88	10	1200	E	19.06
81	13.33	362	A	15.49	13	534	A	16.35	15	552	A	17.80
82	20	1430	U	30.81	12.33	388	A	14.32	8	388	A	10.03
84	10				5	31.8	A	5.38	0.5	129	E	1.42
87	12	865	U	18.52	10	871	U	17.94	5	1010	E	16.74
88	3	582	U	5.88	10	671	U	14.44	10	981	U	17.72
93	4				4	1180	E	14.28	4			
95	20				10	398	A	12.76	4	379	E	7.75
97	7.33	506	U	11.45	10	314	A	11.22	12.5	382	A	13.01
99	7.33	863	U	14.03	1.33	0	A	1.33	0	0	A	0.00
100	33.33	1760	U	48.64	11	829	U	17.99	0			
102	7.33	1770	E	23.86	9.33	1420	E	25.27	16			
105	4	490	E	8.45	2.83	138	U	3.95	2	294	E	4.37
106	10	315	A	11.28	10	728	U	14.56	10	910	U	16.78
108	3.67	345	U	6.31	4.33	723	E	10.49	6	342	A	9.16
109	7	473	U	11.66	0.33	776	E	7.52	0	289	E	2.47
114	0	11.3	A	0.12	0	0	A	0.00	0	0	A	0.00
115	0	826	E	5.74	0	331	E	4.20	0	367	E	3.02
116	8	1970	E	22.07	1.83	860	E	8.90	10	1100	E	19.71

**Table 3.** Average numbers of adjusted cigarettes in each category over trimesters using group-correction method.

***Method***	***Trimester***	***Category***			
		***over reporter***	***accurate reporter***	***under reporter***	***extreme under reporter***
MC	1	-	1.70	6.38	9.07
MC	2	−3.54	1.75	6.77	8.56
MC	3	-	1.21	6.65	7.75
Det	1		1.36	5.69	8.32
Det	2	−3.67	1.49	6.07	7.84
Det	3		0.73	6.06	7.09

**Table 4.** The ratio is the women’s cotinine levels divided by their weighted number of self-reported cigarettes. This ratio shows how much of urinary cotinine a cigarette produces according to women’s self-reports. This table shows the means and the standard deviation for each classification in each trimester.

***Category***	***Trimester 1***		***Trimester 2***		***Trimester 3***	

	***Mean ratio***	***SD ratio***	***Mean ratio***	***SD ratio***	***Mean ratio***	***SD ratio***

Over reporter	-	-	16.24	-	-	-
Accurate reporter	309.79	251.84	278.01	153.03	207.21	152.77
Under reporter	1005.53	2486.65	577.44	579.49	895.17	1002.75
Extreme under reporter	7829.90	21275.18	4740.71	10678.09	2169.90	3019.95
Total	2883.77	11869.41	1434.85	5272.85	1085.01	1941.18

**Table 5.** The average numbers of adjusted cigarettes in each category over trimesters, under different values of cotinine half-life.

***Trimester***	***Category***				
	***Half-life mean***	***over reporter***	***accurate reporter***	***under reporter***	***extreme under reporter***

1	5 hours	-	1.65	6.16	9.14
2	5 hours	-	1.41	6.81	8.60
3	5 hours	-	1.10	6.88	7.92

1	20 hours	-	1.76	6.87	8.75
2	20 hours	−3.47	1.84	6.46	8.88
3	20 hours	-	1.41	5.79	8.42

**Table 6.** The number of women classified into each group in each trimester, using different values of urinary cotinine a cigarette produces. Rows 1–3 use 100 ng/mL, rows 4–6 use 150 ng/mL, and rows 7–9 use 300 ng/mL.

***Cotinine/Cigarette (ng/mL)***	***Trimester***	***Over reporters***	***Accurate reporters***	***Under reporters***	***Extreme under reporters***

100	1	0	10	16	12
100	2	1	17	15	12
100	3	0	13	13	15

150	1	0	21	10	7
150	2	1	25	8	11
150	3	2	15	15	9

300	1	1	30	5	2
300	2	1	35	5	4
300	3	2	30	5	4

## Appendix: Calculations of Uncertainty in Cotinine-Nicotine Cigarette Relationship

We used the following values for the distributions in our Monte Carlo simulation:

Cotinine blood half-life: Normally distributed with mean 527 min, and standard deviation of 100 min.

Ratio Blood-Urine: Normally distributed with mean 8 and standard deviation of 2

Blood cotinine/cigarette: Normally distributed with mean 8, and standard deviation of 0.5.

We derived these values following a set of equilibrium cotinine clearance equations and parameter ranges given to us by Prof. Benowitz’ lab:
Nicotine intake per cigarette = [Cotinine concentration] x [Cotinine clearance] / [f] which is equivalent to:
Blood cotinine concentration per cigarette = (Nicotine per cigarette) / (Cotinine clearance), where:

- Nicotine per cigarette is distributed normally with mean 1.2 mg/cig and range of 0.5 to 2.
- Cotinine clearance per kg of body weight has mean of 1.46 ranging from 0.9 to 2.
- *f* is a scalar with mean 0.78 and ranging from 0.69 to 0.87.

In our calculations we assume the average body weight is 80 kilograms.

Urine cotinine levels that are used to define smokers in the paper are 30 ng/mL. Notice that historically these values were different. For non-pregnant population, in Jarvis *et al.* UK study from the 80's the blood cotinine level used to define smokers was 14 ng/mL (implying urine level of 112 ng/mL). However, in a more recent study, Benowitz and colleagues have found the urinary cutoff-level of less than 0.2 ng/mL [28]. These differences are primarily due to changes in environmental tobacco smoke exposure that have resulted from changes in lifestyle over time and differences in cultural habits as well as the introduction of Indoor Air regulation in the early 90's. Time and cultural trend must be taken into account in studies of smoking nondisclosure during pregnancy: the study we are using was done in the US in mid 90's. It is also important to take into account the number of smokers in the household, since their cutoff for ETS will most likely need to be higher. Notice, however, that for our purposes identifying the exact cutoff for defining a smoker is not very relevant. Using a cutoff lower than 30 ng/mL would have resulted in an inclusion of a subset of women who did not report being a smoker but whose true exposure would be very low (estimated at most 0.5 cigarette per day). This would have negligible effect on our results.
